# Targeted Ablation of Oligodendrocytes Triggers Axonal Damage

**DOI:** 10.1371/journal.pone.0022735

**Published:** 2011-07-27

**Authors:** Aniket Ghosh, Natalia Manrique-Hoyos, Aaron Voigt, Jörg B. Schulz, Mario Kreutzfeldt, Doron Merkler, Mikael Simons

**Affiliations:** 1 Max-Planck-Institute of Experimental Medicine, Göttingen, Germany; 2 Department of Neurology, University of Göttingen, Göttingen, Germany; 3 Department of Neurology and JARA Brain, University Hospital and Medical School, RWTH Aachen, Aachen, Germany; 4 Department of Neuropathology, University Medical Center, Georg August University, Göttingen, Germany; 5 Division of Clinical Pathology, Department of Pathology and Immunology, Geneva Faculty of Medicine, Geneva University Hospital, Geneva, Switzerland; Mental Health Research Institute of Victoria, Australia

## Abstract

Glial dysfunction has been implicated in a number of neurodegenerative diseases. In this study we investigated the consequences of glial and oligodendrocyte ablation on neuronal integrity and survival in *Drosophila* and adult mice, respectively. Targeted genetic ablation of glia was achieved in the adult *Drosophila* nervous system using the GAL80-GAL4 system. In mice, oligodendrocytes were depleted by the injection of diphtheria toxin in MOGi-Cre/iDTR double transgenic animals. Acute depletion of oligodendrocytes induced axonal injury, but did not cause neuronal cell death in mice. Ablation of glia in adult flies triggered neuronal apoptosis and resulted in a marked reduction in motor performance and lifespan. Our study shows that the targeted depletion of glia triggers secondary neurotoxicity and underscores the central contribution of glia to neuronal homeostasis. The models used in this study provide valuable systems for the investigation of therapeutic strategies to prevent axonal or neuronal damage.

## Introduction

All complex nervous systems consist of two main cell types, neurons and glia. The increasing complexity of the nervous system during evolution is accompanied by a steady rise in glial cell number. Whereas only approximately 10% of the 90 000 cells of the central nervous system (CNS) of *Drosophila melanogaster* are of glial origin, glia account for the majority of the cells of the mammalian brain [1]. In vertebrates central nervous system there are three major classes of glial cells: oligodendrocytes, astrocytes and microglia. In both the invertebrate and the vertebrate nervous system, glia participate in an intimate anatomical relationship with neurons to provide structural and metabolic support [2], [3], [4], [5], [6], [7], [8], [9], [10]. There are numerous examples of how glia engage in neurotransmitter metabolisms, ion buffering, axon pathfinding, electrical insulation and nutrient function. These multiple functional interconnections between glia and neurons, raise the question whether and how the acute loss of glia affect neuronal integrity. In *Drosophila*, the glial-specific homeodomain protein, Repo, is essential for survival of the laminar neurons in the optic lobe [11]. Moreover, mutations in genes such as *drop dead* and *swiss cheese*, have revealed the importance of glia in the functioning of the adult *Drosophila* nervous system [12]. In addition, when glial function is impaired in the developing nervous system of *Drosophila* either by mutations in the gene *glial cells missing* or by targeted genetic glial ablation, neuronal death is induced non-autonomously [13]. In mammalian cell culture, astrocytes are required for long-term survival of neurons [14]. Furthermore, several oligodendrocyte mouse mutants result in defects causing axonal degeneration in the CNS [15], [16], [17], [18], [19], [20], [21], [22], [23]. Together, these studies unambiguously show that glial dysfunction can result in neurodegeneration. However, none of these studies have addressed whether the acute depletion of glia in the mature nervous system affects neuronal survival. This is a relevant question as loss of glia in the adult nervous system occurs in a number of neurological diseases. For example, in multiple sclerosis (MS) it is still a matter of debate whether neurodegeneration is the result of a direct inflammatory attack against the axon or rather a consequence of oligodendrocyte dysfunction and demyelination [24], [25], [26], [27]. Especially the latter scenario is difficult to address in the experimental autoimmune encephalomyelitis (EAE) model, the most widely used animal model of MS. To determine whether selective loss of glia in adult brain can cause acute neurodegeneration, we used an experimental system of genetic models that allow the ablation of glia in the adult nervous system of *Drosophila* or mice.

## Results

### Oligodendrocyte ablation in MOGi-Cre/iDTR mice induces axonal damage

**Figure 1:**
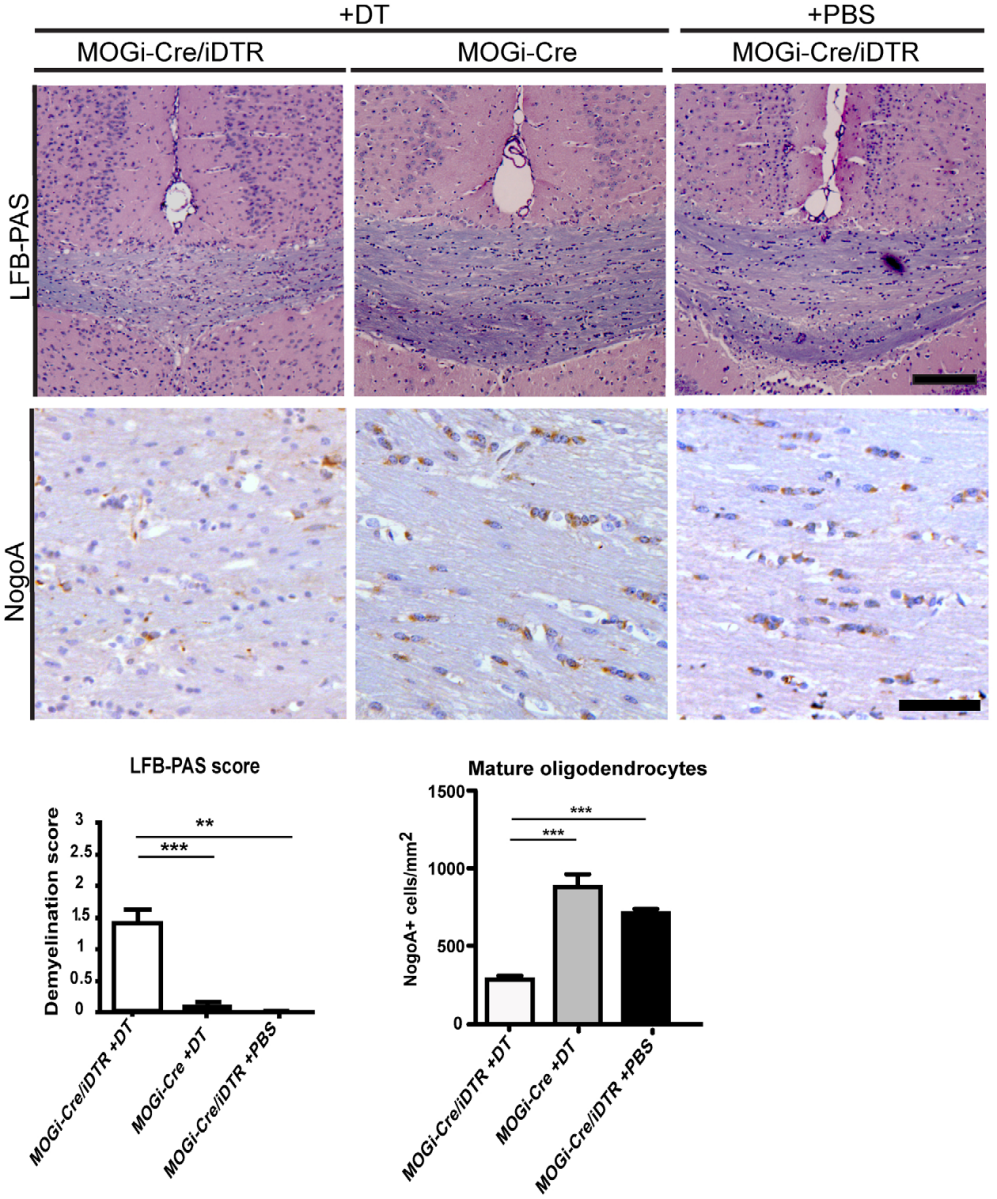
Oligodendrocyte depletion with Diphtheria Toxin in MOGi-Cre/iDTR mice. MOGi-Cre/iDTR mice were injected with 400 ng diphtheria toxin (DT) daily, for 7 days. After 30 days, the level of demyelination was assessed by LFB-PAS staining, and the number of mature oligodendrocytes, by NogoA staining. A decrease of myelination and of mature oligodendrocyte number was observed in MOGi-Cre/iDTR mice treated with DT (left panel), compared to DT-treated MOGi-Cre animals as control (middle panel) and MOGi-Cre/iDTR animals treated with PBS (right panel). For quantification one-way ANOVA was performed, followed by pairwise Tukey test. Statistical significance is represented with asterisks (n = 3–9, **p<0.01, ***p<0.001). Scale bar, 100 µm.

**Figure 2:**
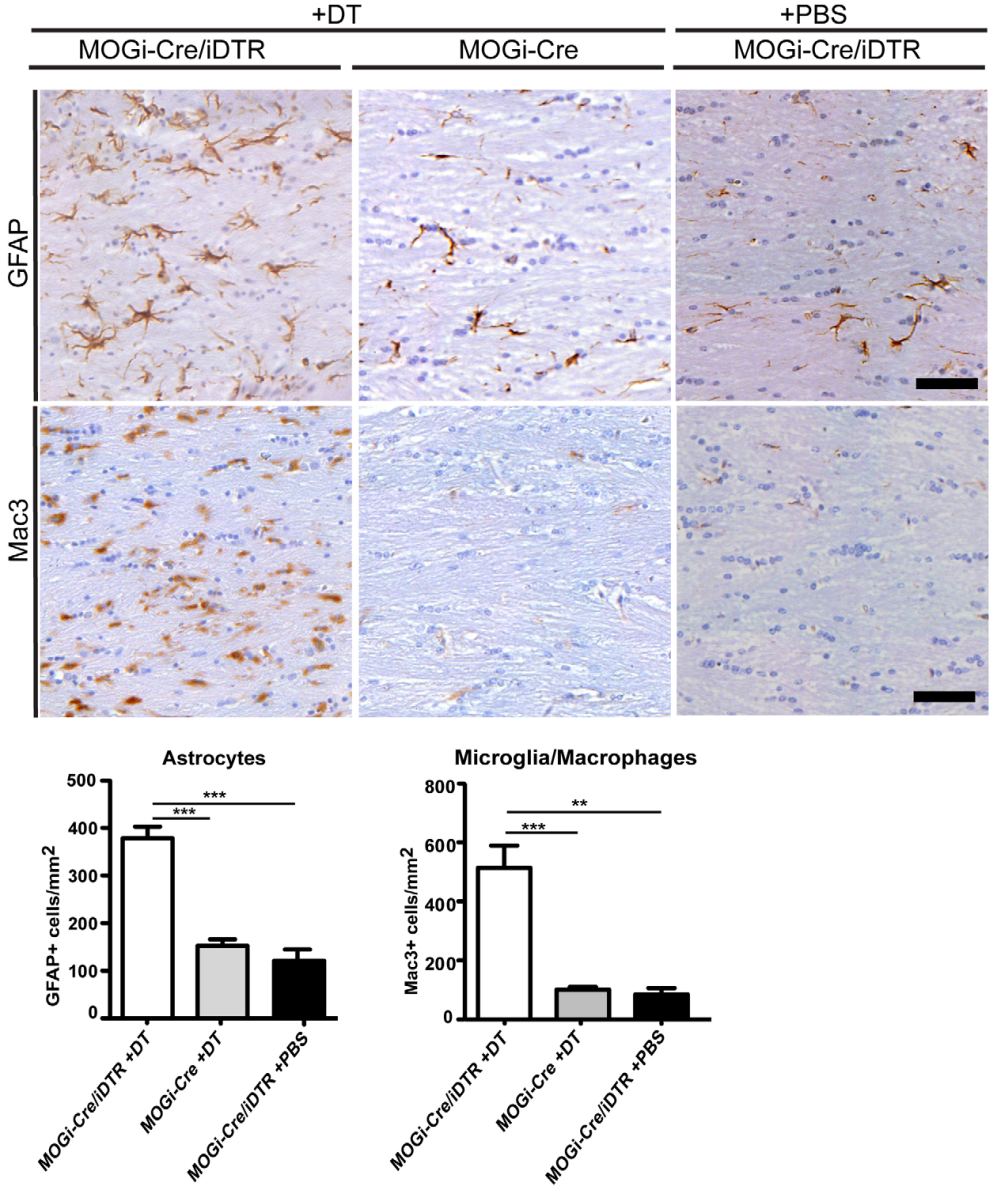
Asrocytosis and microglia activation is observed after DT-induced demyelination in MOGi-Cre/iDTR mice. Coronal sections from MOGi-Cre/iDTR mice treated with DT (left panel), DT-treated MOGi-Cre animals as control (middle panel) and MOGi-Cre/iDTR animals treated with PBS (right panel) were stained for astrocytes (GFAP) and microglia/macrophages (Mac3) 30 days after injection. Quantification of astrocyte and microglia/macrophage density in central corpus callosum is shown as mean ± SEM (n = 3–7). If ANOVA indicated significant differences in the main effect (p<0.05), Tukey test pairwise comparison was applied. Statistically significant differences are indicated by asterisks (**p<0.01, ***p<0.001). Scale bar: 50 µm.

For oligodendrocyte ablation, we used the double transgenic MOGi-Cre/iDTR mice that express the diphtheria toxin receptor specifically in oligodendrocytes. Previous studies have shown that intraperitoneal injection of DT into MOGi-Cre/iDTR mice results in loss of oligodendrocytes and demyelination after −30 days [28]. However, the effect on axonal and neuronal number was not investigated in this study. To explore this issue, 10-week-old MOGi-Cre/iDTR mice received 400 ng of DT via daily intraperitoneal injections on seven consecutive days. Age- and sex-matched MOGi-Cre/iDTR mice that received PBS injections and MOGi-Cre mice (lacking the iDTR allele) that received DT injections served as controls in these experiments. After 30 days, when clinical symptoms such as tremor and unbalanced gait were detected in the treated group, animals were sedated, perfused transcardially, fixed and processed for immunohistochemistry. To analyze the extent of demyelination in the corpus callosum caused by oligodendrocyte ablation, coronal slices were stained with Luxol fast blue–periodic acid Schiff (LFB-PAS) and demyelination was evaluated according to a standardized score from 0 (no demyelination) to 3 (complete demyelination). Consistent demyelination was observed in DT-treated MOGi-Cre/iDTR mice, but was absent in the control animals. In line with this finding, a lower number of oligodendrocytes were observed in DT-treated MOGi-Cre/iDTR mice (Fig. 1). In addition, reactive astrogliosis and activated microglia were detected in DT-treated MOGi-Cre/iDTR mice (Fig. 2).

**Figure 3:**
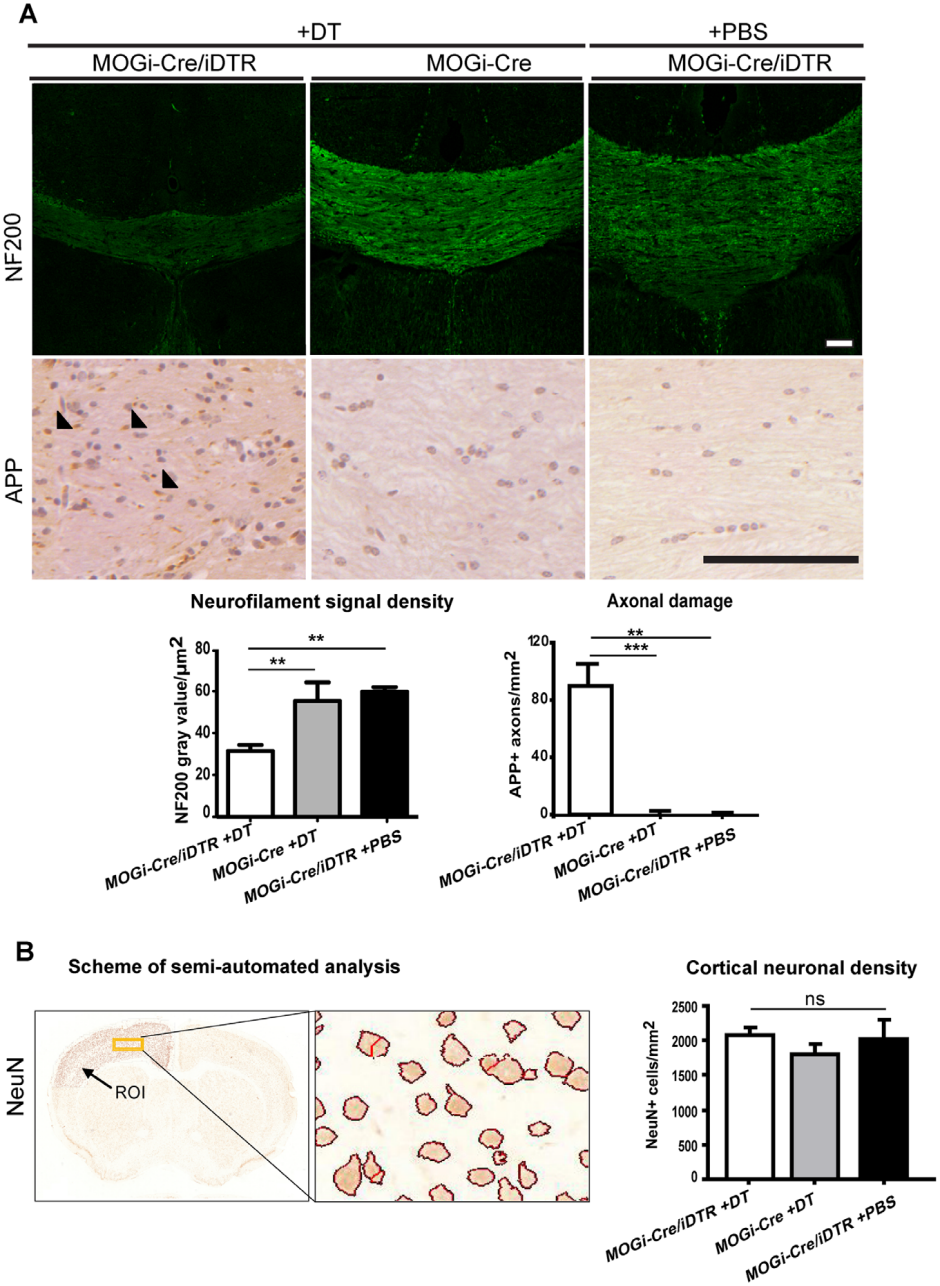
Oligodendrocyte depletion with DT results in axonal damage but does not affect cortical neuronal density. (A) A decrease of neurofilament signal density (NF200), and an increase in axonal damage (evidenced by APP accumulation) was observed in MOGi-Cre/iDTR mice treated with DT (left panel), compared to DT-treated MOGi-Cre animals as control (middle panel) and MOGi-Cre/iDTR animals treated with PBS (right panel). Quantifications are shown as mean and SEM (n = 3–10). One way ANOVA was performed, followed by pairwise Tukey test. Significance is represented with asterisks (**p<0.01, ***p<0.001). Scale bar, 100 µm. (B) Coronal sections were stained for neuronal nuclei with NeuN antibodies, scanned with Mirax Midi System and automatically counted using Definiens Developer XD software in the indicated region of interest (ROI). One way ANOVA was performed, no statistically significant difference in neuronal density was observed between the groups. Also, no significant difference was found in cortical volume between the groups (p>0.05). An average of 10104 NeuN+ were counted per animal. Quantifications represent mean and SEM (n = 8 MOGi-Cre/iDTR mice treated with DT, 5 DT-treated MOGi-Cre animals as control (middle panel) and 3 MOGi-Cre/iDTR animals treated with PBS, ns: not significant).

Next, sections were stained for neurofilament 200 (NF200) to evaluate axonal structures. Images of the midsagittal line of the corpus callosum from coronal sections were obtained under equal acquisition parameters using a confocal microscope and the signal intensity was analysed with Image J. We observed substantial damage of axons in DT-treated MOGi-Cre/iDTR mice as compared to the controls (Fig. 3). In damaged or transected axons, transported molecules and organelles accumulate in the axon and consequently increased amounts of amyloid precursor protein (APP) are frequently detected [27], [29]. We therefore analyzed the axonal pathology in more detail, by performing immunostainings for APP. These experiments revealed a substantial accumulation of APP in the axons of DT-treated MOGi-Cre/iDTR mice, but not in controls (Fig. 3). To determine whether neuronal loss had occurred, we performed a computer-assisted analysis of neuronal densities in defined cortical areas: Sections immunostained with the neuronal marker NeuN were scanned and neuronal densities were evaluated. We found that oligodendrocyte ablation did not result in any significant differences in neuronal cell number (Fig. 3), nor densities. In addition, we could not detect apoptotic neuronal cells by fluorescein-12-deoxy-UTP nick-end labeling (TUNEL) staining (Fig. S1). Together, these results indicate that acute depletion of oligodendrocytes induced axonal injury, but does not cause detectable neuronal cell death 30 days post treatment.

### Glial ablation triggers neuronal apoptosis in adult *Drosophila*

Next, we used *Drosophila melanogaster* as a model system to analyze the impact of glia ablation on neurons in an invertebrate system. Approximately 10% of the cells of the adult *Drosophila melanogaster* CNS are of glial origin. To ablate these cells, we used the UAS/GAL4-system [30]. Glial-specific expression of UAS-transgenes was mediated using the *repo*-*GAL4* driver [31], [32]. The driver line *repo-GAL4* is active already in early fly development. In order to prevent GAL4 dependent expression of UAS-transgenes in development, we made use of a temperature-sensitive allele of the GAL4 repressor GAL80^ts^ [33]. At restrictive temperature (18°C), GAL80 efficiently suppressed GAL4-dependent activation of UAS-transgenes. Shifting adult flies to permissive temperature (29°C), GAL80^ts^ looses its suppressor activity and GAL4 induces the expression of genes under UAS control. To ablate glia, we induced apoptosis specifically in glia by expressing the proapoptotic gene *reaper* in the adult fly using the *repo-GAL4* driver in combination with GAL80^ts^.

**Figure 4:**
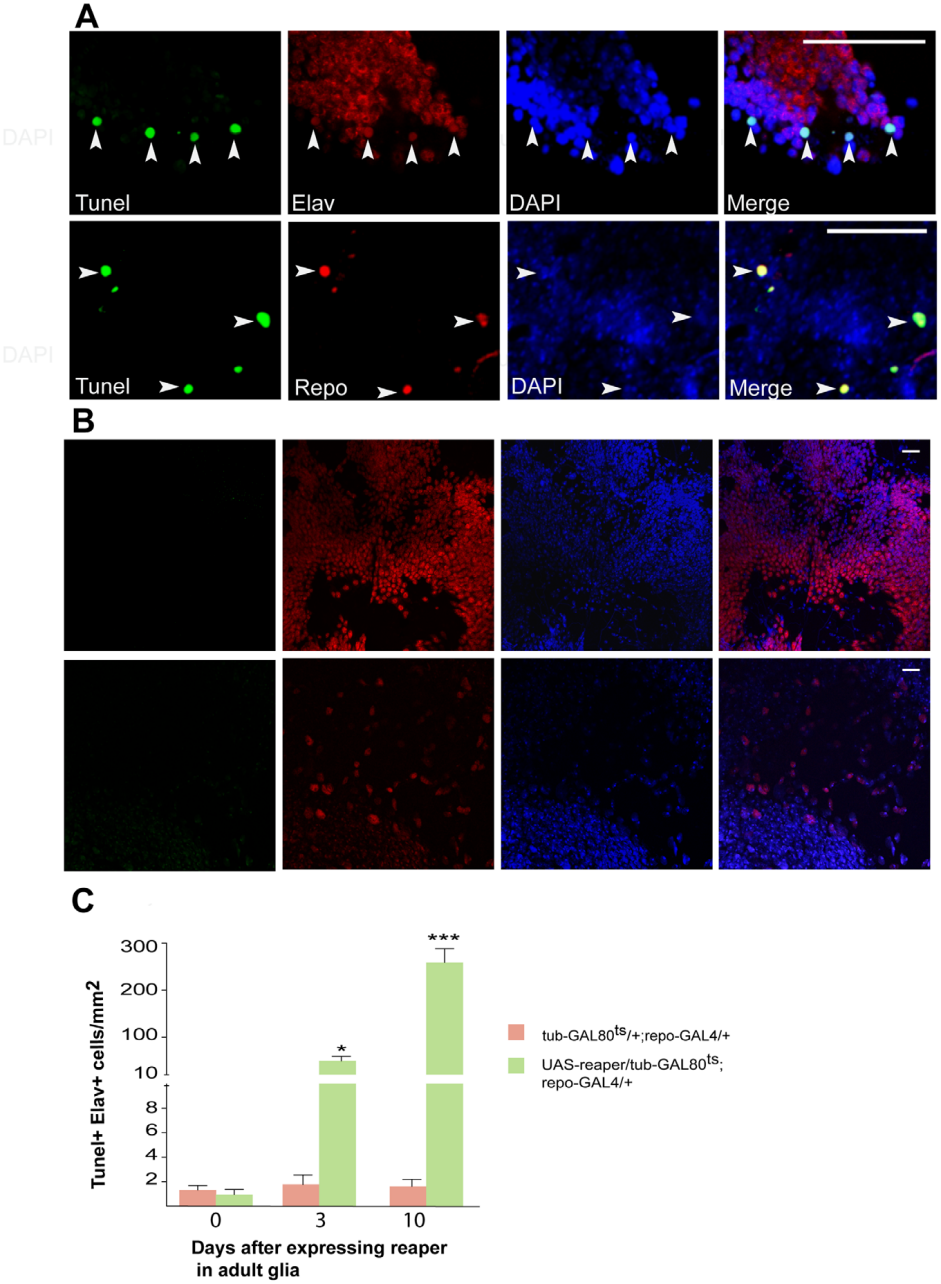
Glial ablation in adult *Drosophila* results in neuronal apoptosis. (A) Glial cell death was induced in transgenic flies by expressing *Reaper* for 10 days in adult flies (*UAS–reaper/tub–GAL80^ts^*;*repo–GAL4/+*. Double-label immunofluorescence and TUNEL staining on brain sections. Colocalization of the glial-specific protein repo and the neuronal-specific protein elav (red) with TUNEL-positive nuclei (green) reveals the presence of apoptotic glial and neuronal nuclei (arrows). (B) Double-label immunofluorescence and TUNEL on brain of control flies (genotype: *tub–GAL80^ts^*/+; *repo–GAL4/+*). Scale bar, 20 µm. (C) Quantitative analysis of TUNEL-positive neurons. Statistical analysis was performed with an unpaired *t* test (***p<0.001, Error bar is ± SEM).

**Figure 5:**
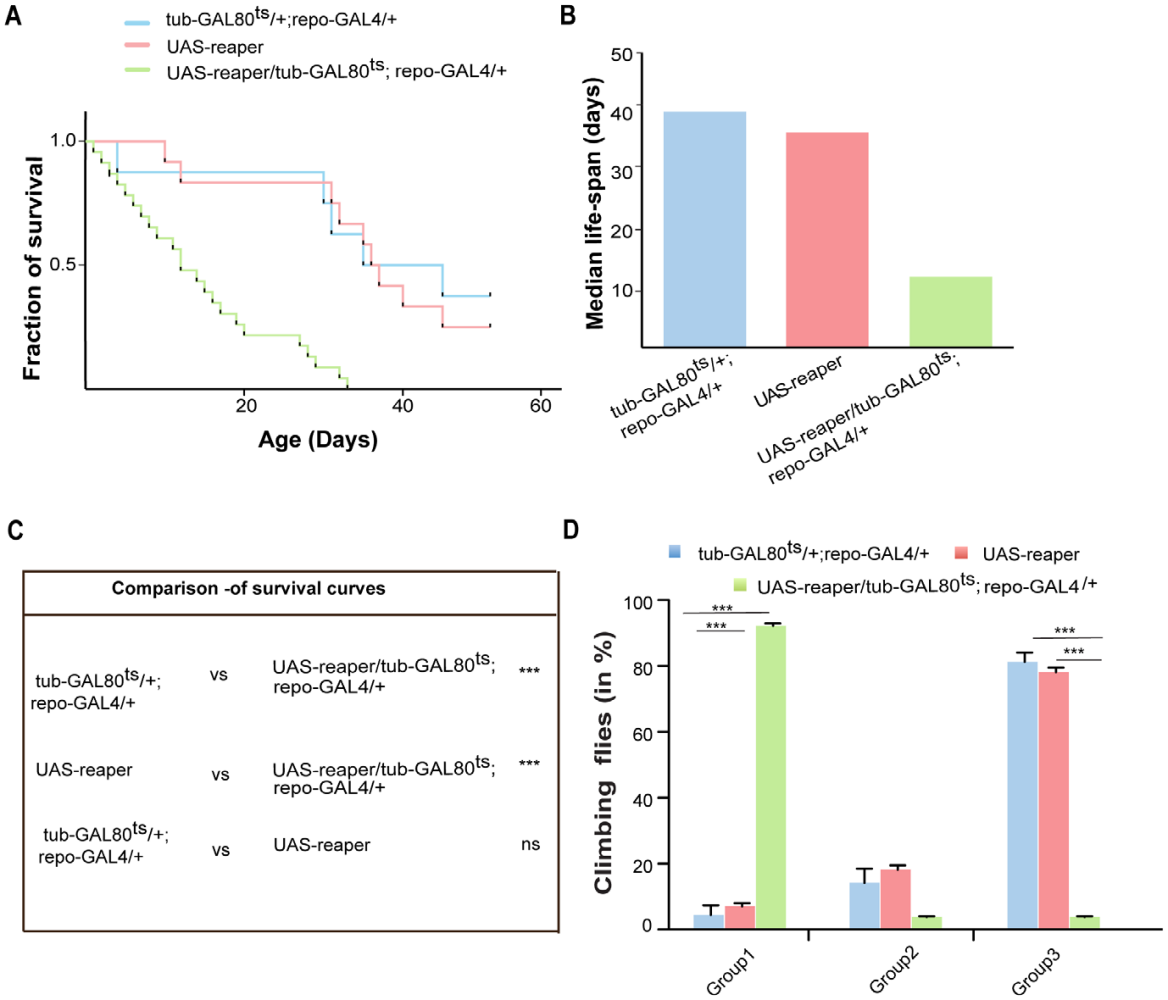
Glial ablation in adult *Drosophila* reduces lifespan and impairs locomotion. (A) Glial cell death was induced in transgenic flies by expressing reaper for 10 days in adult flies (*UAS–reaper/tub–GAL80^ts^*;*repo–GAL4/+*). UAS-reaper and *tub–GAL80^ts^*/+;*repo–GAL4/+* were used as negative control. (B) Median survival of respective survival curve. (C) Summary of statistical significance (Log-rank-Mantel-Cox Test) by cross-comparing the survival curves. (D) Locomotion defect of flies was analyzed with negative geotaxis and was quantified 10 days after shifting the flies from 18°C to 29°C in a countercurrent apparatus. Experimental flies (*UAS–reaper/tub–GAL80^ts^*; *repo–GAL4/+*) were compared to control flies (*tub–GAL80^ts^*/+; *repo–GAL4*/+) in group1 and group3 to have better assessment of motor defect. One-way ANOVA followed by Bonferroni *post hoc* test was used for statistical significance (*p<0.05, **p<0.001, ***p<0.0001, ns not significant).

We performed TUNEL staining and immunofluorescence analyses using antibodies against neuronal and glial markers to detect apoptotic cells and observed massive induction of neuronal apoptosis after triggering cell death in glia (Fig. 4). Quantitative analysis of brain sections examined 0, 3 and 10 days after induction of glial apoptosis, revealed a significant increase in number of TUNEL-positive neurons (Elav-positive cells). Apoptotic neurons were almost absent from control brains. To examine the consequences of glial cell death on the survival of the flies, we performed longevity analysis. After the induction of glial cell death, there was a striking reduction in the lifespan of adult flies as compared to control flies. In addition, flies display negative geotaxis, an easy measure of the ability to climb away from gravity. These experiments revealed dramatic climbing deficits after glia ablation (Fig. 5). Taken together, our results show that the induction of apoptosis in glia of adult flies has a dramatic impact on neuronal survival.

## Discussion

We have shown that selective depletion of glia in the adult vertebrate and invertebrate nervous system results in secondary severe axonal damage or neuronal loss. Targeted genetic ablation of glia was achieved in the adult *Drosophila* nervous system using the UAS/GAL4 system in combination with the temperature sensitive suppressor of GAL4, GAL80^ts^. In mice, oligodendrocytes were depleted by the injection of DT into MOGi-Cre/iDTR double transgenic mice. In both experimental systems the acute depletion of glia induced massive neurotoxicity. These findings are of relevance for interpretation of neurodegeneration in diseases with glial pathology. For example, in patients with MS the neurodegeneration is thought to be the leading cause for permanent disability, but the mechanisms that trigger neurodegeneration are still under debate [27], [34]. Two major – but mutually not excluding – scenarios could be envisaged. An inflammatory response consisting of autoreactive CD8^+^ T cells, antibodies and complement or activated microglia/macrophages may directly attack the axons [35]. Alternatively, axonal damage might be secondary to demyelination. It has been difficult to distinguish between these different possibilities in EAE. Recently, a genetic model that enables the ablation of oligodendrocytes in the mouse nervous system was described. By using an inducible Cre/loxP recombination system, the A subunit of diphtheria toxin was specifically expressed in oligodendrocytes [36]. Interestingly and in contrast to our study, the oligodendrocyte ablation, and the resulting demyelination, did not induce axonal loss in the optic nerve or spinal cord. The reason for these different outcomes is likely to be found in the nature of the different models. The induction of the A subunit of diphtheria toxin in oligodendrocytes by tamoxifen treatment of *PLP/CreER^T^*; *ROSA26-eGFP-DTA* mice may result in a less efficient and less extensive ablation as compared to the approach used in our study. While our paper was in revision, Pohl et al. [37] presented another model of oligodendrocyte depletion using a tamoxifen (TAM)-dependent *PLPCreERT2* allele in combination with a Cre-dependent diphtheria toxin fragment A (DT-A) transgene in the ubiquitously expressed *ROSA26* locus. In this study, in coherence with our results, axonal damage was observed as detected by a reduction in neurofilament staining intensity and an increase in APP accumulation compared to control.

These findings indicate that the nature of the oligodendrocyte attack has major influence on the response of the axons. It is possible that the rapid or widespread death of oligodendrocytes overwhelms the defence mechanisms of the brain. There are a number of oligodendrocyte mouse mutants that result in axonal degeneration, but some of them require several months to develop robust degeneration. For example, mouse mutants that lack some of the major myelin genes such as myelin-associated glycoprotein (MAG), the 2′,3′- cyclic nucleotide 3′- phoshodiesterase (CNP) and the proteolipid protein (PLP) have an almost normal live span and develop late-onset, chronic progressive axonal degeneration [15], [16], [38]. Interestingly, the neuronal pathology in MS seems also to be primarily directed against the axon, as axonal loss is more frequently found than loss of neuronal cell bodies [27]. However, loss of cortical neurons has recently been reported in more advanced stages of the disease so called secondary progressive MS [39].

In contrast, the acute depletion of glia in the adult *Drosophila* nervous system triggered apoptosis in neurons, motor paralysis and death within a few days. Even if glia account for only ∼10% of the cells in the adult *Drosophila* nervous system, it is likely that their ablation compromise the physiological environment of the CNS to an extent that can not be compensated by the neurons. However, when comparing the results between the *Drosophila* and the mice models, it is important to note that we used the pan-glial driver *repo* in the flies, whereas only oligodendrocytes were ablated in the mice. It is therefore possible that a more widespread ablation of glia would also result in apoptosis and an acute loss of neurons in mice as observed in *Drosophila*. Such changes in the physiological milieu of the CNS induced by glial dysfunction might be relevant for a number of neurological diseases. For example, models of diverse human neurodegenerative diseases, including amyotrophic lateral sclerosis (ALS), Huntington's disease and multiple system atrophy (MSA) provide evidence that there are non-cell-autonomous mechanisms in which neurodegeneration is strongly influenced by dysfunctional glia [40], [41], [42], [43], [44].

Together these studies indicate that glial dysfunction can trigger neurotoxicity in a number of different ways. Our study shows that the acute ablation of glia has an immediate impact on neuronal or axonal viability. The results provide evidence that loss of oligodendrocytes is sufficient to induce acute axonal damage and underscores the central contribution of glia to neurodegenerative diseases. Many previous studies have related demyelination with an axonal pathology that does not necessarily result in actual axonal loss. In fact after transection in demyelinating diseases an axon can degenerate while the neuronal cell body is able to survive [27], [45]. More research into the non-inflammatory mechanisms of neurodegeneration will open new avenues for the treatment of diseases such as MS. Importantly, the model used in this study provides a valuable system for the investigation of therapeutic strategies to prevent axonal or neuronal damage.

## Materials and Methods

### Mice and perfusions

Animal experiments were conducted in accordance with animal protection laws approved by the Government of Lower Saxony, Germany. 10-week-old MOGi-Cre/iDTR mice [46], [47] were injected intraperitoneally with 400 ng of diphtheria toxin (DT, Merck) in PBS once a day for seven days. Age- and sex-matched MOGi-Cre/iDTR mice injected with PBS, and MOGi-Cre mice (lacking the iDTR allele) injected with DT were used as controls. After 30 days, when clinical symptoms such as tremor and unbalanced gait were detected in the treated group, animals were sedated with a 14% chloral hydrate intraperitoneal injection, perfused transcardially and fixed with 4% paraformaldehyde (PFA). Tissue was processed as described previously [48], [49]. After paraffin embedding 3 µm thick sections of the brain, spinal cord, spleen and liver were obtained.

### Histological analysis

The extent of demyelination induced by DT injection was assessed by scoring the animals from 0 (no demyelination) to 3 (complete demyelination) in sections stained with Luxol fast blue–periodic acid Schiff (LFB–PAS) [50] by a double-blinded observer. Immunohistochemistry was performed using antibodies against neuronal nuclei (NeuN Chemicon, Temecula, CA, USA) and against the amyloid precursor protein (APP, clone 22C11; Chemicon, CA, USA) followed by labelling with biotinylated secondary antibodies. Avidin-biotin technique with 3,3-diaminobenzidine was used for visualization. Fluorescence immunohistochemistry was performed for neurofilament 200 (NF200, clone N52, Sigma) with Alexa488-conjugated chicken anti-mouse IgG secondary antibodies (Invitrogen). To assess axonal preservation, images of each side of the midsagittal line of the corpus callosum from coronal sections stained with NF200 were obtained under equal acquisition parameters with a confocal microscope (LSM 510, Carl Zeiss MicroImaging, Inc). At each side of the midsagittal line, the NF200-positive area (which allows the demarcation of the corpus callosum and its distinction from the ventricular and cortical areas), was taken as ROI and analyzed with imageJ. The signal density is given by the average gray value per square micrometer of the ROI, which is independent of surface area.

Histological sections stained with anti-APP counterstained with Haemalaun, and with NeuN antibody were scanned using the Mirax Midi System (Carl Zeiss Micro Imaging GmbH).

### Neuronal numbers semi-automated analysis

Sections stained for neuronal nuclei with NeuN antibodies were scanned as described above. Neuronal numbers were automatically counted using a script in Cognition Network Language based upon the Definiens Cognition Network Technology® platform (Definiens Developer XD software, Munich, Germany). Briefly, the cortical region of interest was drawn manually and NeuN positive cells were detected based on color criteria. After finer segmentation to discriminate between nucleus and cytoplasm, the object was classified as a NeuN-positive cell if the soma was below a certain size and had 0 or 1 nucleus. If more than one nucleus was detected within one soma, the object was split using each nucleus as seed to grow into the surrounding cytoplasm, stopping if growing borders converged or the cytoplasmic border was reached. Finally, the total number and density of NeuN-positive cells was calculated.

### *Drosophila* stocks and genetics

The following fly stocks were used: w[1118]; P{w[+m*] = GAL4}repo/TM3 referred to as *repo–GAL4* in text, w[*]; P{w[+mC] = tubP-GAL80[ts]}, referred to as tub-GAL80^ts^, in text, w[1118]; P{w[+mC] = UAS-rpr.C}, referred to as UAS-reaper in text. OregonR served as wild-type control. All stocks were obtained from the Bloomington Stock Center (Indiana University, Bloomington, IN USA). Flies were maintained on normal commensal-yeast-agar medium.

By combining glial specific driver repo-GAL4 [31] with ubiquitously expressed temperature-sensitive allele of GAL80^ts^ we generated a fly line (w; tub-GAL80^ts^; repo-GAL4/TM3, Sb) referred to as tub-GAL80^ts^; repo-GAL4 [33], allowing expression of reaper [51] specifically in adult glia. Crossings of UAS-reaper with tub-GAL80^ts^; repo-GAL4 flies were set at 18°C to minimize the expression of reaper. At 18°C the ubiquitously expressed GAL80^ts^ blocks repo-GAL4-dependent activation UAS-reaper. From the F1 generation adult flies genotype of the following genotypes UAS-reaper/tub-GAL80^ts^;repo-GAL4/+ and tub-GAL80^ts^/+;repo-GAL4/+ (control) were switched to 29°C to inactivate the temperature-sensitive GAL80, thereby allowing the expression *reaper* in glia. We used this system to ablate adult glia in *Drosophila*.

### Longevity assay

To deplete the mature glial cells tub-GAL80^ts^; repo-GAL4 flies were crossed with UAS-reaper, and OregonR (negative control). 3–4 days post-hatching adult males with the respective combination of GAL4-driver, UAS-transgene and GAL80^ts^ were shifted to 29°C (10–15 flies per vial). Numbers of dead flies were counted daily. Fresh fly food was provided every 2–3 days. At least 50 flies per genotype were used for the assay. Log Rank Test (Mantel-Cox) was used to test for statistical significance.

### Climbing Assay

30–40 male flies per genotype were shifted to 29°C (3–4 days post-hatching). 10 days after shifting to 29°C negative geotaxis assay was performed to asses locomotion. In this assay, flies were partitioned up into six tubes by giving them the choice five times to stay or to climb up the side of the tube. After the assay, flies were distributed into six tubes depending on how many times (between 0 and 5 times) they climbed up. To represent the distribution of the flies, the number of the flies in the 1st and 2nd (group 1), 3rd and 4th (group 2), and 5th and 6th (group 3) tubes were summed up and displayed in a bar graph [51].

### TUNEL Assay

To detect apoptotic cells, *in situ* cell death detection kit from Roche was used and performed according to manufacturer protocol. To stop the terminal deoxynucleotidyl transferase-mediated biotinylated UTP nick end labelling (TUNEL) reaction saline-sodium citrate buffer (Promega) was used.

### Immunohistochemistry

Adult *Drosophila* brains were dissected in 1× PBS +0.1% Triton X-100 (PBT) and fixed with 4% PFA for 30 min at room temperature. Primary antibodies (both obtained from Developmental Studies Hybridoma Bank) anti-Repo (1∶100) and anti-Elav (1∶200) were diluted in PBT with 2% horse serum and incubated at 4°C overnight. For primary antibody detection, Cy3-coupled antibodies anti mouse or rat (Dianova) were used in a 1∶200 dilution, respectively. After extensive washing, brains were mounted with Vectashield (Vectorlab)+ DAPI and images were taken with a confocal microscope (Leica LSM/SP2). Central brain region was imaged and analyzed for quantification. Image processing was performed with ImageJ.

### Statistical analysis

For the histological analysis of the diphtheria toxin assay, a one-way ANOVA was performed, followed by a Tukey test for pairwise comparisons, if applicable. For *Drosophila* glia ablation, unpaired t-test was performed for the histological analysis of TUNEL-positive neurons, as well as a Log-rank-Mantel-Cox Test for the cross-comparison of survival curves and one-way ANOVA followed by Bonferroni *post hoc* test for motor defect time course analysis. A p-level of <0.05 was considered significant in all tests. Statistics were calculated using GraphPad prism and SigmaPlot software (Systat software, Inc.).

### Ethics Statement

All animal treatments were approved in advance by the Lower Saxony state authorities (“Niedersächsisches Landesamt für Verbraucherschutz und Lebensmittelsicherheit”; Postfach 39 49; 26029 Oldenburg) for animal experimentation and conducted in accordance with animal protection laws approved by the Government of Lower Saxony, Germany. The Approval ID is: 33.14-42502-04-068/09) and the project name “Mechanismen der chronischen Progredienz bei Multipler Sklerose”).

## Supporting Information

Figure S1 **Coronal sections of cortex from MOGi-Cre/iDTR mice treated with DT (left panel), DT-treated MOGi-Cre animals as control (middle panel) and MOGi-Cre/iDTR animals treated with PBS (right panel) were stained for TUNEL and NeuN 30 days after injection.** Sections of the spleen of the respective groups were used as positive control for the TUNEL stainings. Colocalisation of DAPI and TUNEL-positive cells is shown as an inset for each group. Scale bar: 50 µm, for inset 10 µm.
(DOC)
