# Supplementary material for: Targeted Ablation of Oligodendrocytes Triggers Axonal Damage
Source: PLoS One. 2011 Jul 27;6(7):e22735. doi: 10.1371/journal.pone.0022735 (PMC3144945; doi:10.1371/journal.pone.0022735)
Supplement: Figure S1 — Coronal sections of cortex from MOGi-Cre/iDTR mice treated with DT (left panel), DT-treated MOGi-Cre animals as control (middle panel) and MOGi-Cre/iDTR animals treated with PBS (right panel) were stained for TUNEL and NeuN 30 days after injection. Sections of the spleen of the respective groups were used as positive control for the TUNEL stainings. Colocalisation of DAPI and TUNEL-positive cells is shown as an inset for each group. Scale bar: 50 µm, for inset 10 µm. (DOC) [file pone.0022735.s001.doc]

**
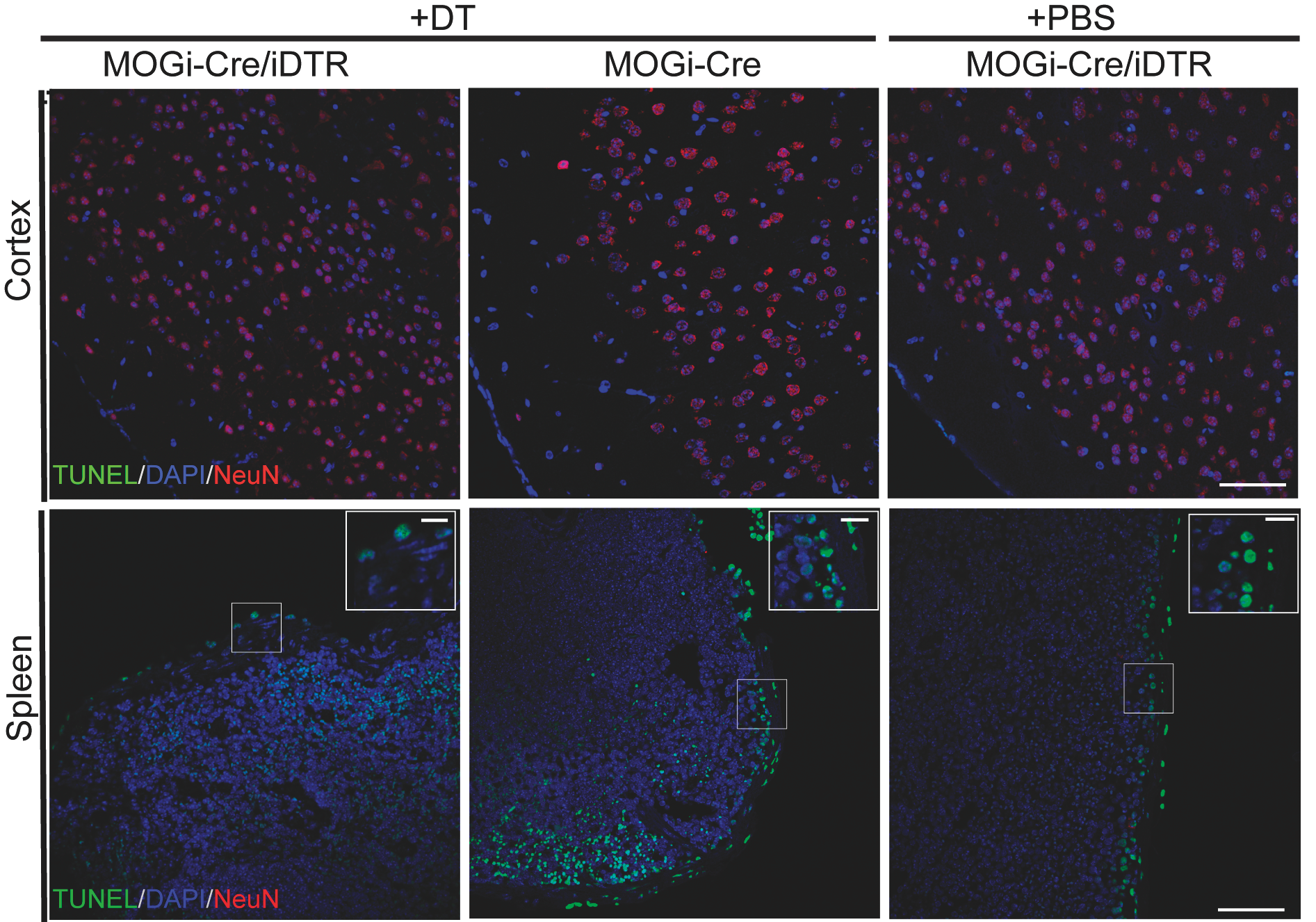
**

**Figure S1**

Coronal sections of cortex from MOGi-Cre/iDTR mice treated with DT (left panel), DT-treated MOGi-Cre animals as control (middle panel) and MOGi-Cre/iDTR animals treated with PBS (right panel) were stained for TUNEL and NeuN 30 days after injection. Sections of the spleen of the respective groups were used as positive control for the TUNEL stainings. Colocalisation of DAPI and TUNEL-positive cells is shown as an inset for each group. Scale bar: 50 µm, for inset 10 µm.
